# The Effects of pH on Orientations of Adsorbed Lysozyme on Negatively Charged Surfaces

**DOI:** 10.1021/acs.jpcb.6c01437

**Published:** 2026-07-22

**Authors:** Dante A. Anhesini, Ricardo B. de Souza Júnior, Daniel L. Z. Caetano, Icaro P. Caruso, Andrey G. Cherstvy, Sidney J. de Carvalho

**Affiliations:** † Department of Physics, 28108 São Paulo State University (UNESP), Institute of Biosciences, Humanities and Exact Sciences, 15054-000 São José do Rio Preto, Brazil; ‡ Institute for Physics & Astronomy, 26583 University of Potsdam, 14476 Potsdam-Golm, Germany

## Abstract

The adsorption of proteins on charged surfaces is essential for a number of applications, such as biosensors and biocatalytic systems. An important aspect here is the orientation of the adsorbed proteins on the surface, determined in part by their pH-dependent patchy charge distribution, which can also be affected by the presence of charged surfaces in proximity (due to the charge-regulation (CR) mechanism). In this study, the hen-egg white lysozyme (HEWL) is adopted as a model protein to study the features of the protein adsorption into a negatively charged surface via employing a coarse-grained model and constant-pH Monte Carlo simulations. Our goal is to study the CR effects on the charge-patch distribution and how this affects the orientation of the adsorbed protein molecules. We observe an orientation transition in which the amino-acid residue HIS15 stops being in contact with the surface and starts being positioned on the opposite protein face, whereas the LYS33 residue undergoes the opposite inversion. This transition takes place at lower pH values, when the protein charges are constant in the course of simulations, because the CR mechanism promotes the persistence of positive-charge patches and the emergence of negative-patches at higher pH values. This promotes the stability of the orientation observed at low pH.

## Graphical abstract

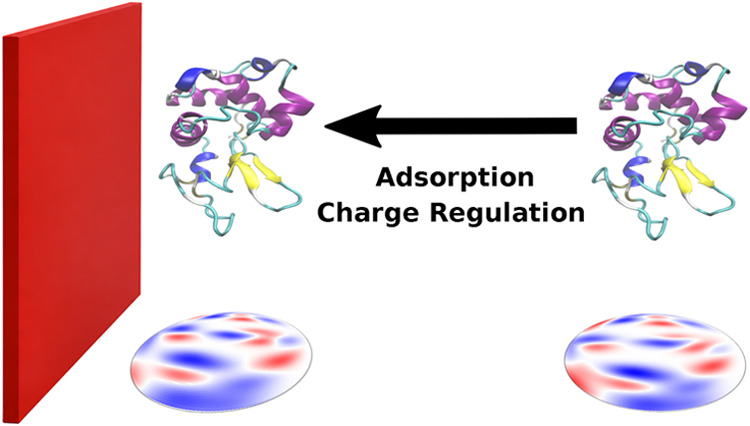

## 1 Introduction

Understanding the physical-chemical mechanisms governing the orientational specificity of proteins adsorbed onto charged surfaces is crucial for the rational design of biosensors, for drug-delivery platforms, as well as for biocatalytic interfaces. In such applications, the preservation of protein functionality crucially depends on the accessibility of specific structural motifs, such as the catalytic or binding sites.^1–4^ The preferential orientation of an adsorbed protein with respect to a given surface arises from its often intrinsically heterogeneous and anisotropic charge distribution. The latter gives rise to localized surface-exposed regions bearing net charges which are opposite in sign to those of the substrate itself. These regions—commonly referred to as “charge patches” ^5,6^ —mediate directional electrostatic (ES) interactions with the surface and provide a dominant, energetically favorable contribution to the adsorption free energy. As a result, these interactions not only drive the adsorption, but also stabilize specific protein orientations by minimizing the ES component of the free energy.

More broadly, the charge patches are localized charge patterns on a particle or surface characterized by their size and charge density.^7–9^ Several studies have investigated various aspects of the effective interactions between such particles and surfaces, including the ES attraction arising from spatial correlations between oppositely charged patches^10,11^ and the modulation of attractive and repulsive contributions determined by the relationship between patch size and the screening length in the solution.^12,13^

Another significant contribution to the free energy of protein adsorption arises from the charge-regulation (CR) mechanism, which originates from fluctuations in the protonation states of ionizable amino-acid (AA) residues. These fluctuations render the residues responsive to the charge-containing environment and allow their protonation probabilities to adjust in the presence of nearby charged entities, such as fixed surfaces or other macromolecules. The CR strength can be quantified by the parameter *c* = ⟨*z* ^2^⟩ – ⟨*z*⟩^2^, commonly referred to as the “capacitance” or CR capacity, where *z* denotes the valence of a given residue.^14–20^ The ES potential generated by the surface—or, equivalently, the variations of the local pH—acts on ionizable residues and can modify not only the net charge of the protein,^21–24^ but also its spatial charge distribution.^25–28^

Such redistribution can in turn significantly enhance the protein–surface interactions by promoting certain ES-favorable orientations. For instance, Bošič and Podgornik demonstrated^28,29^ that within the CR framework, the multipole moments associated with an inhomogeneous protein-charge distribution become coupled in a manner that explicitly depends on the capacitance. Using the Mollweide projections^30^ of the protein-charge density,^27,31^ they showed that it was possible to directly visualize how the pH influences the charge-multipole moments^27^ and also to identify which protein regions are most strongly affected by the CR effects.^28^

Recently, the adsorption of hen egg-white lysozyme (HEWL) inside a charged pore was investigated by some of the current authors using a coarse-grained protein model combined with the constant-pH Monte Carlo simulations.^25^ HEWL has been extensively employed as a benchmark system in several studies of protein adsorption onto charged surfaces.^32–35^ HEWL is also known to exhibit antimicrobial activity,^36,37^ which can be significantly enhanced upon *protein immobilization* on various solid supports.^38^ It was shown^25^ that increasing the pH toward the isoelectric point (pI) of HEWL induces a transition in the preferred orientation of the adsorbed HEWL protein. By mapping the ES potential on the protein surface for each orientational state, it was demonstrated^25^ that the CR mechanism drives substantial changes in both the local charge density and the spatial distribution on the protein’s adsorbing face. These CR-induced rearrangements favor the adsorption by stabilizing distinct orientational states through the ES interactions.

Despite these findings, a fundamental question remains open: how does a coupling between the ionizable AA residues, inherently introduced by the CR mechanism, determine the formation and the spatial organization of the charge patches on the protein surface? Moreover, how does this CR-mediated patchiness influence the protein orientation upon its adsorption, in comparison to the models assuming a fixed, patchy distribution of charges without any CR?

In this work, we revisit the adsorption of the HEWL onto a flat negatively charged surface, focusing on the role of pH in modulating the distribution of charge patches on the protein surface. In particular, we investigate how the variations in the inhomogeneous surface-charge density induced by the CR mechanism govern the protein-orientation transitions, as we previously reported in ref 25. By systematically comparing the results of computer simulations obtained with and without the CR effects, we assess the relative importance of the interplay of the CR mechanism and the effects of intrinsic charge patchiness onto the protein adsorption.

The paper is organized as follows. In Section 2, we describe the protein model, the computational simulation framework, and the methodology employed to map the protein charge density via a Mollweide projection. In Section 3, we analyze the pH dependence of the protein orientation and discuss the results in terms of the CR-driven charge-patch distributions. Finally, in Section 4, we summarize the main findings and outline some potential applications of the present results.

## 2 Methods

### 2.1 Model and Simulation

We adopt below a coarse-grained model of a single HEWL protein placed in the vicinity of a flat charged surface. The HEWL modeling is based on the same “Structure-Based Model”, as we previously employed,^25^ so that each AA residue is represented by a single sphere centered at the C_α_ position,^40–44^ see Figure 1. If an AA residue is ionizable, its charge is located at the center of the sphere. This centrally charged single-bead model was used in our previous studies^25,42–44^ and was shown to provide an adequate description of the systems under investigation. More detailed coarse-grained models, in which the charges of amino acid residues are displaced from the C_α_ position to the side chain, have also been reported in the literature. Such models have proven suitable for describing salt effects on protein folding,^45^ the behavior of intrinsically disordered protein ensembles,^46,47^ and some features of the protein–protein interactions.^48,49^

**Figure 1:**
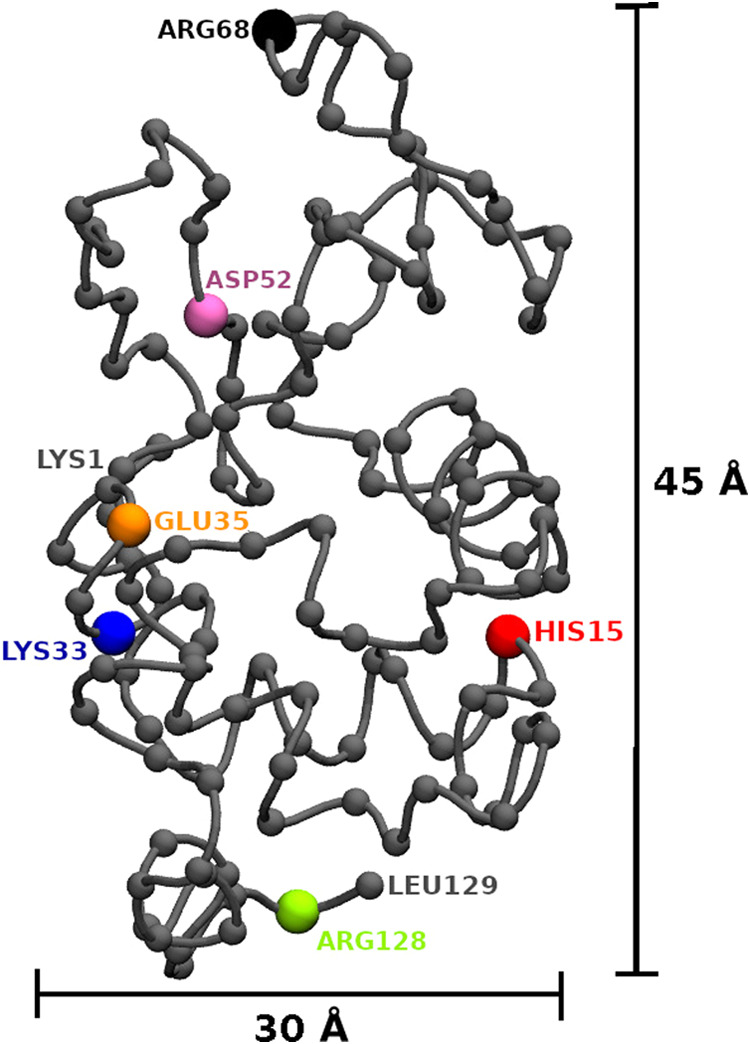
Structure-based model representation of the HEWL protein constructed from the PDB structure (PDB ID is 3WUN). Each AA residue is modeled as a single sphere centered at the C_α_ position. The residues HIS15 (red), LYS33 (blue), GLU35 (orange), ASP52 (pink), ARG68 (black), and ARG128 (yellow) are highlighted, with the residues GLU35 and ASP52 constituting the active site of the HEWL. Under the standard biological conditions (i.e., at pH = 7 and for the ionic strength of 0.1 M), the net charge of HEWL is +8*e*_0_, where *e*_0_ denotes the elementary electric charge, and its isoelectric point is ≈11, as obtained from the titration curve presented in ref 39. The terminal HEWL residues LYS1 and LEU129 are indicated, alongside with the HEWL approximate physical dimensions.

The energy of a given protein conformation can be written as (in units of *k*_B_ *T* ≡ β^–1^)­

1 $$\beta E=\sum_{\mathrm{bonds}}\left(100\;{Å}^{-2}\right)\times \xi {\left(r-r_{0}\right)}^{2}+\sum_{\mathrm{angles}}20\times \xi {\left(\theta -\theta_{0}\right)}^{2},+\sum_{\mathrm{dihedrals}}\xi \left\{\left[1-\cos\left(\phi -\phi_{0}\right)\right]+\left[1-\cos\left(3\left(\phi -\phi_{0}\right)\right)\right]/2\right\},+\sum_{\mathrm{contacts}}\xi \left[5{\left(d_{\mathrm{ij}}/r_{\mathrm{ij}}\right)}^{12}-6{\left(d_{\mathrm{ij}}/r_{\mathrm{ij}}\right)}^{10}\right],+\sum_{\mathrm{non}‐\mathrm{contacts}}\xi {\left(d_{nc}/r_{\mathrm{ij}}\right)}^{12},+\sum_{\mathrm{charges}}\left[z_{i}z_{j}l_{B}\exp\left(-\kappa r_{\mathrm{ij}}\right)/r_{\mathrm{ij}}+z_{i}V_{s}\left(x_{i}\right)\right]$$

Here, *r*, θ, and ϕ denote, respectively, the distances between two bonded AA residues, the bond angles formed by three consecutive residues, and the dihedral angles defined by four consecutive residues, while *r*_*ij*_ represents the distance between the *i*th and *j*th residues. The reference values of *r*_0_, θ_0_, and ϕ_0_ are obtained from the native structure of HEWL provided in the PDB file with the ID 3WUN. The parameter *d*_*ij*_ corresponds to the distance between the residues *i* and *j* which are in contact in the native structure, determined by the so-called “Contact of Structural Units” contact map,^50^ whereas *d*_*nc*_ = 4.0 Å characterizes the repulsive interactions between those AA residues which are not in the native contact. The first five non-ES terms in eq 1 depend on the parameter ξ = 1.06 *k*_B_ *T* adopted from ref 25 to reproduce the experimental melting temperature^51^ of the HEWL molecule. The numerical prefactors multiplying ξ in the first two terms are chosen to ensure the appropriate balance between the energetic contributions associated with the tertiary contacts and those arising from the torsional degrees of freedom, as described in detail in ref 40. In the following, other forces such as the hydrophobic and fluctuation-induced interactions are neglected, with the main focus being on the implications of the ES forces dominating the behavior of our model system at realistic conditions.

### 2.2 ES Potential and Titration

The last two terms in eq 1 account for the ES interactions.^42–44^ In these terms, *z*_*i*_ and *z*_*j*_ denote the valencies of the AA residues *i* and *j*, respectively, and *l*_B_ ≈ 7.1 Å is the Bjerrum length. The quantity *V*_s_(*x*_*i*_) represents the ES potential (in units of *k*_B_ *T*/*e*_0_) at a distance *x*_*i*_ from the surface, corresponding to the position of residue *i*. The parameter κ^–1^ is the ES screening length defined as^52^

2 $$\kappa =\sqrt{8\pi l_{B}n_{0}}$$

where *n*_0_ is the bulk number density of ions of a 1:1 salt constituting the electrolyte solution of our model.

We consider surface charge density σ_s_ of a planar surface depends on the solution pH, thereby modeling the experimentally measured charge density of silica nanoparticle.^53^ The values of σ_s_ as a function of pH for the salt concentrations adopted in this work are shown in Figure 2. As these surface charge densities can generate surface ES potentials exceeding approximately 25 mV,^54,55^ the *linearized* PB approximation may introduce significant inaccuracies in the ES potential values near the charged interfaces. Therefore, the ES potential at a distance *x* from the surface is obtained by solving the full *nonlinear* Poisson–Boltzmann equation, with solution for the ES potential given by^54,56^

3 $$V_{s}\left(x\right)=2\ln\left[\frac{1+\tanh\left(V_{0}/4\right)\exp\left(-\kappa x\right)}{1-\tanh\left(V_{0}/4\right)\exp\left(-\kappa x\right)}\right]$$

Here, the dependence of the ES potential at the surface, *V*_0_ = *V*_s_(0), with the surface-charge number density σ_s_ (in units of Å^–2^) is described by the Grahame equation^54,56^ via

4 $$\sigma_{s}=\sqrt{\frac{2n_{0}}{\pi l_{B}}}\sinh\left(\frac{V_{0}}{2}\right)$$

Note that in eq 1 only the direct ES interactions^57,58^ are included, while possible image-force ES repulsions occurring upon adsorption onto low-dielectric surfaces are neglected, see ref 15 for more theoretical details.

**Figure 2:**
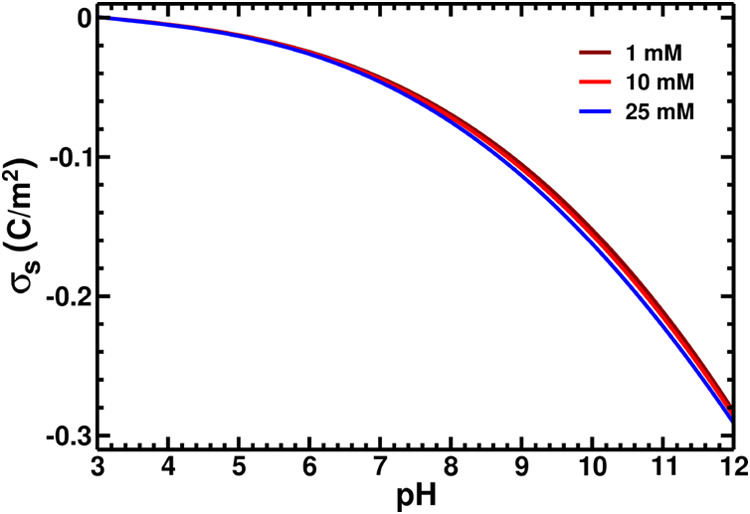
Dependence of the surface charge density σ_*s*_ as a function of pH for a set of salt concentrations used in this work. The values shown were obtained from the function ∑_*n*=0_^4^(*A*_*n*_ *c*_s_ + *B*_*n*_)­pH^*n*^, where *c*_s_ is the salt concentration and the coefficients *A*_*n*_ and *B*_*n*_ are the fits to the experimental data for silica SBA-15 nanoparticles reported in ref 53. For an easier macro-to-micro conversion of physical units, the charge density of 1 C/m^2^ corresponds to 6.242 *e*_0_/nm^2^.

Metropolis Monte Carlo simulations in the canonical ensemble are employed to sample the protein’s configurational space, including its conformations, positions, and orientations relative to the charged surface. The Monte Carlo moves used to update the HEWL configuration are^60^ (i) local displacements of an AA residue; (ii) pivot rotation; (iii) crankshaft rotation; (iv) rigid-body translation; and (v) rotation of the entire protein. At each Monte Carlo step, one of these five moves is randomly attempted.

In addition, in 30% of the Monte Carlo steps, the protonation state of a randomly selected titratable AA residue is allowed to change according to the Metropolis criterion, with the corresponding energy variation given by^42,61^

5 $$\beta \Delta E_{\mathrm{titr}}=\beta \Delta E_{\mathrm{ES}}\pm \eta \log10\times \left(\mathrm{pH}-{pK}_{0}\right)$$

where η = +1 (η = −1) is for a basic (acidic) residue. The positive and negative signs in eq 5 correspond to the protonation and deprotonation events, respectively. The first term in eq 5 represents the change in the ES energy associated with the protonation or deprotonation. The second term in eq 5 corresponds to the contribution of the compound model, in which p*K*_0_ is the intrinsic (i.e., in the absence of the ES interactions) dissociation constant of the AA residue,^25,62^ as reported in Table 1. Only those cysteine residues that are not involved in the disulfide bonds are allowed to titrate in the current model.^59^ Although each HEWL molecule contains eight cysteine residues, all of them participate in disulfide bridges and are, therefore, treated as *charge-neutral* in the present model.

**Table 1:** Titratable AA Residues of the HEWL, as Identified from the Structural Analysis of its PDB Data

Residue	Type	Occurrence	p*K*_0_
ASP	Acidic	7	4.0
GLU	Acidic	2	4.5
TYR	Acidic	3	9.6
CYS^a^	Acidic	0	10.8
HIS	Basic	1	6.3
LYS	Basic	6	10.6
ARG	Basic	11	12.0
Total number	–	30	–

a Cysteine is a titratable residue only in its free form.^59^

As our primary objective here is to assess the effect of pH on the orientation of HEWL adsorbed onto a charged surface, we model the protein-charge distribution using two distinct approaches. In the *first approach*, the average charges of AA residues are calculated for a protein molecule being free in solution. These average values are then assigned as the fixed charges during the simulations of this protein in the presence of a charged surface. Hereafter, this approach is referred to as the fixed-charges (FC) model.

In the *second approach*, the simulations are initiated with the HEWL in the presence of the charged surface, with all titratable AA residues initially set in their charged states. The protonation states of these residues are subsequently allowed to change/fluctuate according to eq 5, and the protein undergoes an equilibration stage at the beginning of the simulation (see below), ensuring that the final results are independent of the initial protein charge state and its spatial configuration. This second approach is hereafter called the CR model.

For most of the results below, we use a low monovalent-salt concentration of 0.001 M corresponding to *n*_0_ = 6.022 × 10^–7^ Å^–3^ in order to enhance the ES effects (a so-called weak-screening regime). The salt effect was verified by adopting additional concentrations of 0.01 and 0.025 M. The solution pH is varied from 5 to 14, encompassing both the isoelectric point of HEWL (pI ≈ 11)^39^ and the pH range over which HEWL exhibits antimicrobial activity, approximately from pH = 6 to pH = 9, with the optimal activity around pH ≈ 7.4.^36^ Note that the surface adsorption is a commonly employed strategy to extend the lifetime of HEWL, which is desirable for its application as an antimicrobial agent.^38^ The temperature is kept fixed at *T* = 298.15 K. Under these conditions, the HEWL protein remains stable and folded, exhibiting a small root-mean-square deviation and a well-defined mean-square radius of gyration, see ref 25 for further details. An equilibration stage consisting of 10^6^ Monte Carlo steps is performed in the simulations, while the average properties are computed from 10^7^ statistically uncorrelated protein configurations.

We highlight that the CR in the present constant-pH Monte Carlo framework extends beyond a simple modulation of the net protein charge. As the protonation equilibria are dynamically coupled to the local ES environment generated by the charged surface, ionic screening, and protein orientation, different adsorption geometries expose distinct titratable residues to different ES conditions. Consequently, the adsorption orientation influences the protonation probabilities, while the resulting charge redistribution alters the ES adsorption free energy, generating a feedback mechanism between the protonation equilibrium and orientational selection. This adaptive response modifies not only the effective total charge of the protein, but also its anisotropic ES character, implicitly affecting its effective multipolar properties. Although an explicit decomposition into the dipolar or higher-order multipole moments was not pursued in the present study, such effects are naturally embedded in the constant-pH sampling of protonation states available.

### 2.3 Surface-Charge-Density Projection

Given a pronounced heterogeneity of the protein-charge distributions and to provide a clearer visualization of the pH-dependent formation of the charge patches, we adopt the approach proposed by Božič and Podgornik in refs 27,28,31 . In this framework, a continuous spherical surface-charge density is constructed from the discrete protein charges using the von Mises-Fisher distribution.^31,63^ Briefly, the spherical surface-charge density σ­(θ, ϕ) is obtained by projecting all *N* protein charges onto a spherical surface of radius *R* that encloses the entire protein charge distribution. The contribution of each charge is evaluated at a point {θ, ϕ} on the sphere, yielding a continuous representation of the protein surface charge-number density. Accordingly, σ­(θ, ϕ) is given by^31^

6 $$\sigma \left(\theta ,\phi \right)=\frac{1}{4\pi R^{2}}\sum_{k=1}^{N}\frac{z_{k}\lambda_{k}\exp\left[\lambda_{k}\cos\left(\gamma_{k}\right)\right]}{\sinh\left(\lambda_{k}\right)}$$

where the numbers *z*_*k*_ with *k* = {1, 2, ..., *N*} denote the valence of the *k*th protein residue and λ_*k*_ are the parameters that specify how spread out the projection should be.^31^ Moreover, cos γ_*k*_ represents the cosine of the geodesic angle, i.e., the shortest path on the spherical surface between the point {θ, ϕ} and point {θ_*k*_, ϕ_*k*_}, namely^64^

7 $$\cos\left(\gamma_{k}\right)=\cos\left(\theta \right)\cos\left(\theta_{k}\right)+\sin\left(\theta \right)\sin\left(\theta_{k}\right)\cos\left(\phi -\phi_{k}\right)$$

We set below the following parameters: *N* = 30 for the number of protein residues (see also Table 1), λ_*k*_ = 20 for all protein charges, and *R* = 23 Å for the radius of rotation of the HEWL molecule.^65^ Finally, the surface-charge density obtained from eq 6 is mapped from the spherical surface onto a plane using the Mollweide projection.^30,31^

This approach provides an effective visual framework for analyzing various nonhomogeneous charge distributions of the molecules in a single 2D representation, allowing the identification of positive and negative charge patches formed by clustered or isolated charged AA residues on the protein surface.^31^ Similar projection methodologies^66–68^ have been employed to investigate various charge distributions,^27^ to study the protein regions involved in adsorption onto surfaces with different binding affinities,^69^ and to examine the surface properties of proteins.^68^ We apply this method in what follows to assess how the charge patches of the HEWL are modulated by the effects of pH and of CR.

## 3 Results and Discussion

### 3.1 Positions and Orientations of HEWL

We initiate the analysis by identifying the regions and the respective AA residues of HEWL protein that are the closest to the charged surface. We then map the position of the titratable AA residues by computing their probability distribution functions, *P*(*x*), as a function of their distance *x* from the surface. Following the procedure adopted in our previous study,^25^ we divide the space near the surface into three regions—(I) close to the surface (*x* ≤ 10 Å), (II) intermediate separations (10 Å < *x* ≤ 20 Å), and (III) far from the surface (*x* > 20 Å)—to match the estimated region occupied by the adsorbed protein. This choice is based on the protein’s average diameter of ∼30 Å (twice the HEWL radius of gyration of about 15 Å), thereby providing an appropriate spatial framework for analyzing protein orientations through the identification of the residues with a higher probability of occupying each of these three region.

Within the pH range considered in this study, the protein is found to undergo some orientational transitions such that certain AA residues exhibit *bimodal* probability-distribution functions with their peaks located in regions (I) and (III). The residues displaying the most pronounced probability peaks in these regions, as well as clear transitions of these peaks between regions (I) and (III), were selected to characterize the overall protein orientation.

We identify that at pH = 7 the AA residues HIS15 and LYS33 are more likely to be located either in the regions close to the surface (*x* < 10 Å) or far away from it (*x* > 20 Å). Figures 3A and 3B present the probability distribution *P*(*x*) of these two residues, respectively, at pH = 7 (darker lines) and pH = 11 (lighter lines), considering both the CR (dashed lines) and FC (solid lines). Except at pH = 11 with the FC, the results indicate the coexistence of two distinct adsorbed orientational states. At pH = 7, the CR results showed a higher probability for HIS15 to be located in region (I) and for LYS33 to be located in region (III), indicating that the inclusion of the CR promotes a *more directional adsorption* of HEWL. In contrast, at pH = 11, i.e., near the isoelectric point of HEWL (pI ≈ 11), the FC results did not exhibit a stable adsorbed state. Including CR is found to stabilize the adsorption and also to promote a highly directional configuration of HEWL protein, in which LYS33 is preferentially located closer to the surface.

**Figure 3:**
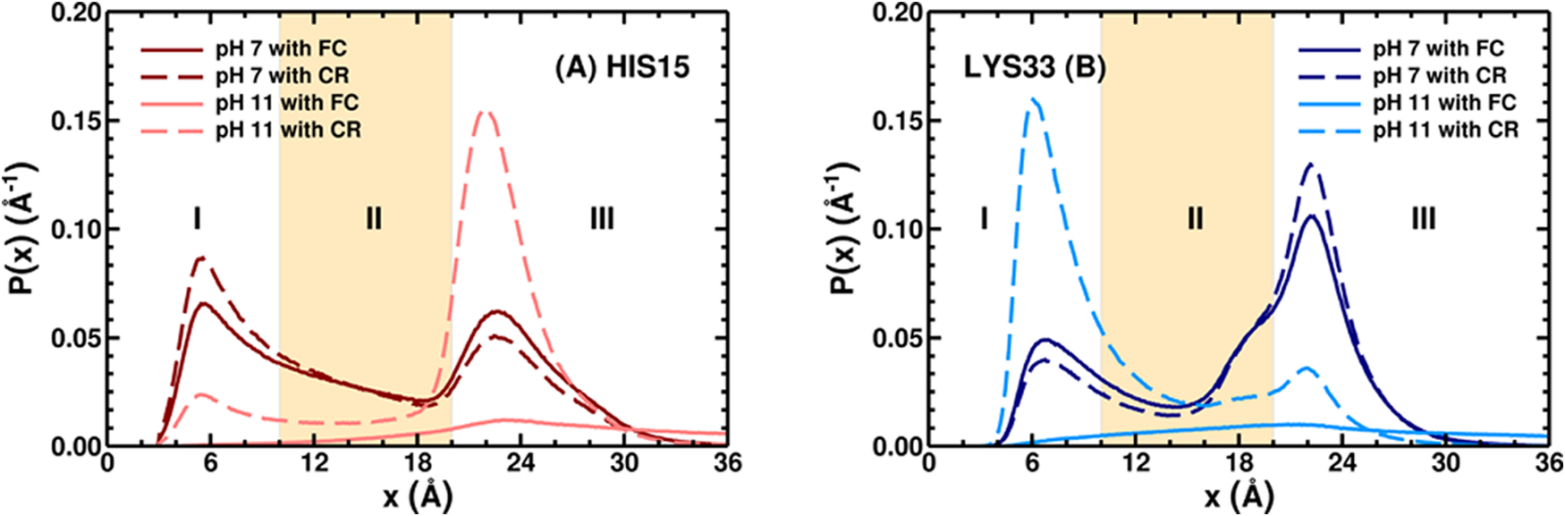
Probability distribution of the AA residues HIS15 (panel (A)) and LYS33 (panel (B)) as a function of the distance *x* from the surface at pH = 7 (the dark red curves in (A) and the navy blue curves in (B)) and pH = 11 (the light red curves in (A) and the light blue curves in (B)). In both panels, the solid (dashed) curves correspond to the simulations performed with the FC (CR) model. The concentration of 1:1 salt is 0.001 M, corresponding to *n*_0_ = 6.022 × 10^–7^ Å^–3^.

Let us compare these findings to some existing experimental evidence. Specifically, Antonov and coauthors^70^ employed electron paramagnetic resonance to investigate the orientation of HEWL on porous oxide carriers, including silica with the pore sizes of the order of hundreds of nanometers. The protein molecules were spin-labeled at the HIS15 residue. For a physical adsorption at pH = 7.15, two distinct binding modes were identified: one site with the HIS15 residue oriented toward the surface and another site where it remained exposed to the solvent. The quantitative analysis of the relative populations of these two modes^70^ reveals a clear preference for the former configuration, in agreement with our results discussed above.

As illustrated in Figure 1, HIS15 and LYS33 are located on the opposite sides of the protein, with the line connecting them defining the *short axis* of the HEWL molecule. The *long axis* is defined by the line connecting the ARG68 and ARG128 residues.^71^ A more global characterization of the protein orientation can be obtained by evaluating the angles being formed by these axes with the surface.^71–75^ Here, these angles are measured using the vectors from LYS33 to HIS15 for the short and from ARG128 to ARG68 for the long axis.

Figures 4A and 4B show the probability distribution *P*(θ) of the angle θ between the long axis and the surface for the FC and CR models, respectively. The insets show the HEWL center-of-mass distance from the surface as a function of pH. In general, for the FC model, the center-of-mass distance remains close to 19 ± 3 Å over the pH range from 6 to 9.5, indicative of stable protein adsorption in this pH range. At pH = 10, the center-of-mass distance increases by about 1 Å and its deviation by about 3 Å, indicating that adsorption is progressively weakened as the net protein charge decreases and the protein charge patches become insufficient to ensure the adsorption.

**Figure 4:**
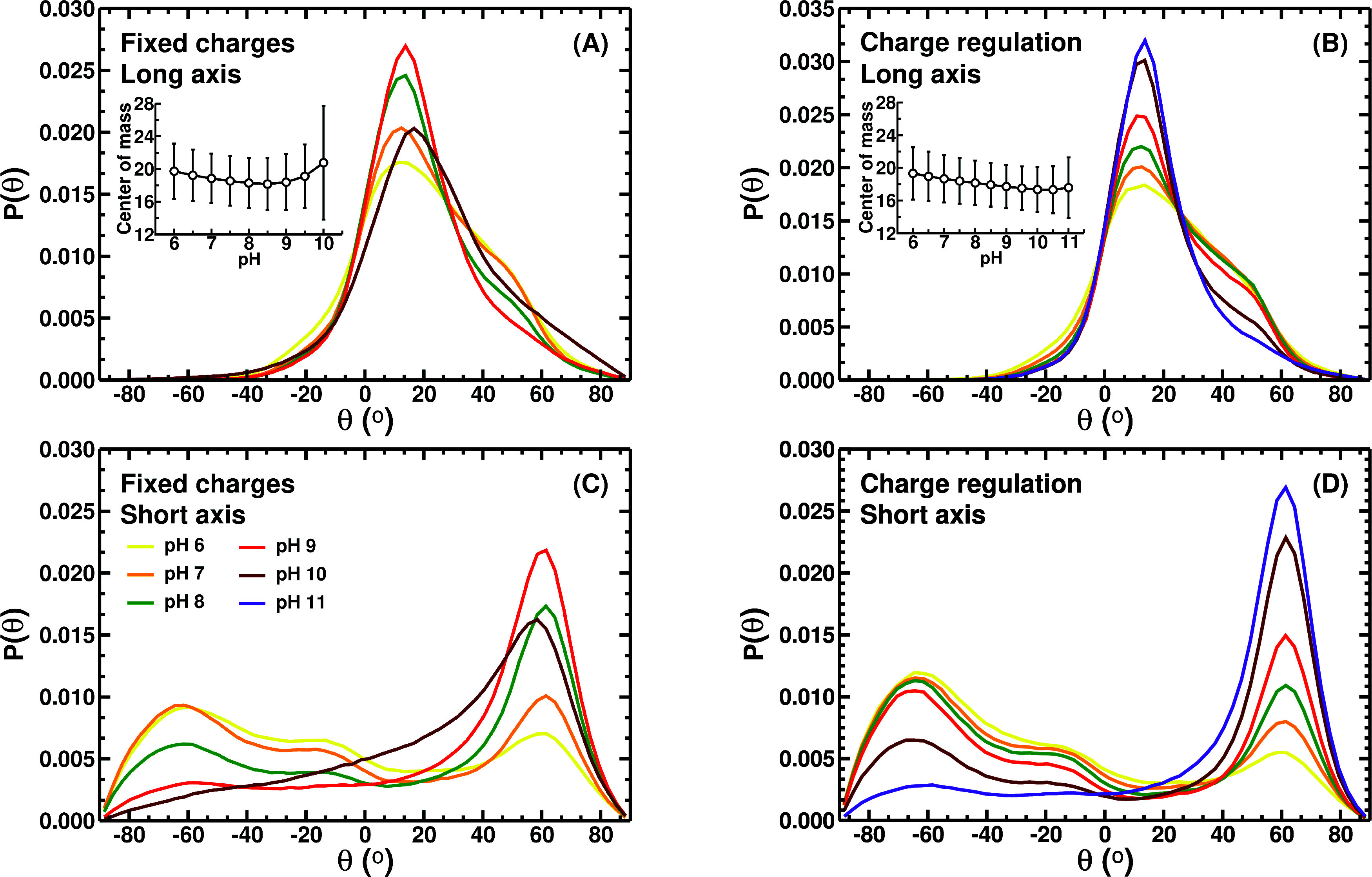
Probability distributions of the HEWL short and long axes as a function of the angle θ between the corresponding axis and the adsorbing surface. Panels (A) and (C) show the FC results, whereas panels (B) and (D) show the CR results. In both cases, the pH values range from 6 to 11. The insets show the protein center-of-mass distance from the surface as a function of pH. The salt concentration is the same as in Figure 3.

When the CR is considered, both the center-of-mass distance from the surface and its deviation are comparatively smaller, as visible from the inset of Figure 4B, confirming that CR favors HEWL adsorption. For both charge models, the *P*(θ) distributions indicate that the most probable orientation corresponds to an angle of ≈12° for the ARG128 → ARG68 vector, suggesting that the long axis of the adsorbed protein lies approximately parallel to the surface. This result is consistent with experimental observations at low surface coverage,^32,76,77^ with ARG128 located slightly closer to the surface. This orientation, in which the long axis of HEWL is approximately parallel to the surface, is referred to as the “side-on” orientation. Conversely, the orientation in which the long axis is approximately perpendicular to the surface is referred to as the “end-on” orientation, whereas intermediate orientations are classified as “between”.^71,74^ There is also a population of configurations in which θ ≈ 50°, which characterizes the “between” orientation and it is consistent with all-atom molecular dynamics simulations from ref 71.

Figures 4C and 4D show the probability distributions for the angle between the short axis and the surface for the FC and CR models, respectively. In general, *P*(θ) displays a *bimodal* behavior, consistent with the distance distributions of HIS15 and LYS33 to the surface shown in Figure 3. At low pH, this coexistence of orientations favors those configurations with θ ≈ −60°, indicating that the protein region containing the HIS15 residue is preferentially located closer to the surface, while LYS33 remains farther away. When the pH is increased, this spatial arrangement is *reversed*, resulting in a most probable value of θ ≈ +60°. As the HEWL protein preserves its native structure under the conditions considered—exhibiting a low root-mean-square deviation (≈1.5 Å) and a nearly constant radius of gyration (≈15 Å)^25^—this inversion can be unambiguously attributed to a change in protein orientation upon adsorption to the surface. The CR also results in smaller fluctuations of θ along both the long and short axes, confirming that CR promotes a more well-defined adsorption orientation.

This pronounced pH-dependent directionality is illustrated in Figures 5A and 5B, which show the population fractions *p*_–_ = ∫_–π/2_^0^ *P*(θ)­d*θ* and *p*_+_ = ∫_0_^π/2^ *P*(θ)­d*θ* associated with the negative- and positive-orientation states, respectively, as a function of solution pH for both the FC and CR cases. Negative-orientation states are represented by the dark red circles, whereas positive-orientation states are represented by the navy blue squares. When the charges of all AA residues are kept constant, the orientational transition of HEWL occurs at pH ≈ 7.5. In contrast, when the CR is taken into account, the orientational inversion shifts to pH ≈ 9.3. Under these conditions, the preferred adsorption orientation remains stable even at pH values above the isoelectric point of HEWL because the charged surface alters the protonation states of the AA residues, thereby enhancing protein–surface interactions and stabilizing the adsorbed state.

**Figure 5:**
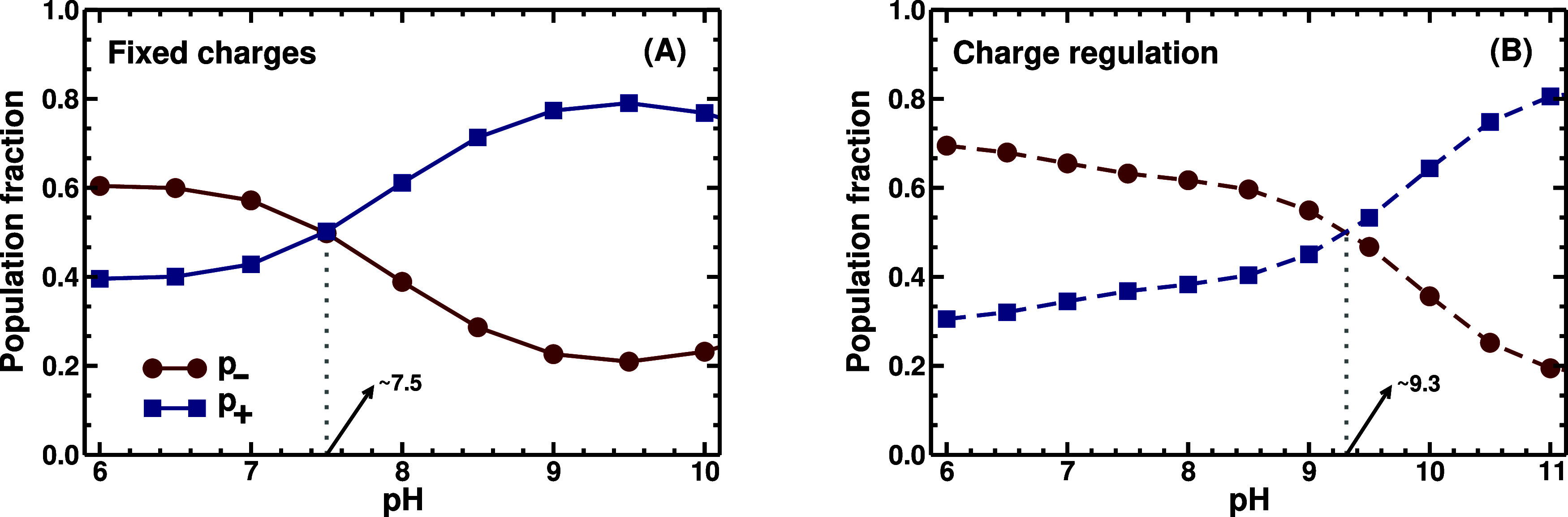
Population fractions *p*_–_ and *p*+ associated with configurations exhibiting negative (dark red circles) and positive (navy blue squares) angles between the short axis and the surface, respectively, as a function of pH. Panel (A) shows the FC results, whereas panel (B) show the CR result. The gray dotted lines indicate the pH values at which the orientational inversion in the protein molecule occurs. The salt concentration is the same as the one used in Figure 3.

Let us discuss now one representative study of HEWL adsorption. Namely, Kubiak-Ossowska and coauthors^71^ investigated the lysozyme adsorption onto a flat negatively charged silica surface using a combination of detailed atomistic molecular dynamics simulations and of multiparametric experiments, including surface-plasmon-resonance, contact-angle, and zeta-potential measurements. They reported that the protein adopts well-defined orientations (“side-on” or “between”) relative to the surface at pH ≤ 10. At higher pH values, the authors of ref 71 concluded that the protein orientation could not be resolved, as adsorption was predominantly driven by the hydrophobic interactions. In this context, our current simulations recover the pH range over which the orientational transition occurs when the CR mechanism is taken into account. Moreover, our results predict the emergence of an additional ES-driven specific orientation of the HEWL protein at high pH, which was not identified in the experimental study.^71^

Let us note shortly why the HEWL orientation on surfaces is important. Studies on HEWL adsorption into confining surfaces—such as those encountered when biomolecules are in contact with porous materials—have attracted considerable attention due to its potential to enhance both protein lifetime and its antimicrobial activity.^38^ In such systems, the adsorbed protein must adopt an orientation in which its active site remains exposed, thereby enabling ligand binding. In its antimicrobial-activity mechanism, the HEWL hydrolyzes several structurally related substrates, most notably alternating polysaccharide copolymers of *N*-acetylglucosamine (NAG) and *N*-acetylmuramic acid (NAM).^36,37,78,79^

### 3.2 Mollweide Projection

Motivated by this, we map the spatial location of the HEWL active site formed by the acidic AA residues GLU35 and ASP52. These residues are located on the same side of the protein as the LYS33 residue (see Figure 1), and therefore their behavior can be assumed to be qualitatively similar. At pH = 7.4, corresponding to the optimal enzymatic activity of HEWL,^36^ both active-site residues remain exposed to the solvent in both the CR and FC simulations. This preferential orientation is consistent with the experimental observations reported by Daly and coauthors^76^ using certain surface-sensitive techniques. Importantly, the HEWL retains significant antimicrobial activity over a broader pH range, for 6 ≲ pH ≲ 9.^36^ Our results indicate that the CR mechanism enables the exposure of the HEWL active site over this entire pH interval, thereby encompassing all the conditions under which the protein exhibits antimicrobial activity.

To elucidate the differences in pH at which the orientational transitions of the adsorbed HEWLs occur, we analyze the surface-charge-density projection of the protein. Figure 6 displays the surface-charge density of HEWL, in the absence of the CR, projected onto a plane using the Mollweide projection^27,28,31^ for pH = 7 and pH = 11, as described in Section 2.3. The AA residues indicated by the arrows in Figure 6 are located closest to the charged surface at each pH value. Increasing the pH from 7 to 11 leads to a shrinkage and a reduction in the intensity of the positively charged patch (shown in blue Figure 6, see also ref 62), mainly due to the effects of lysine deprotonation.

**Figure 6:**
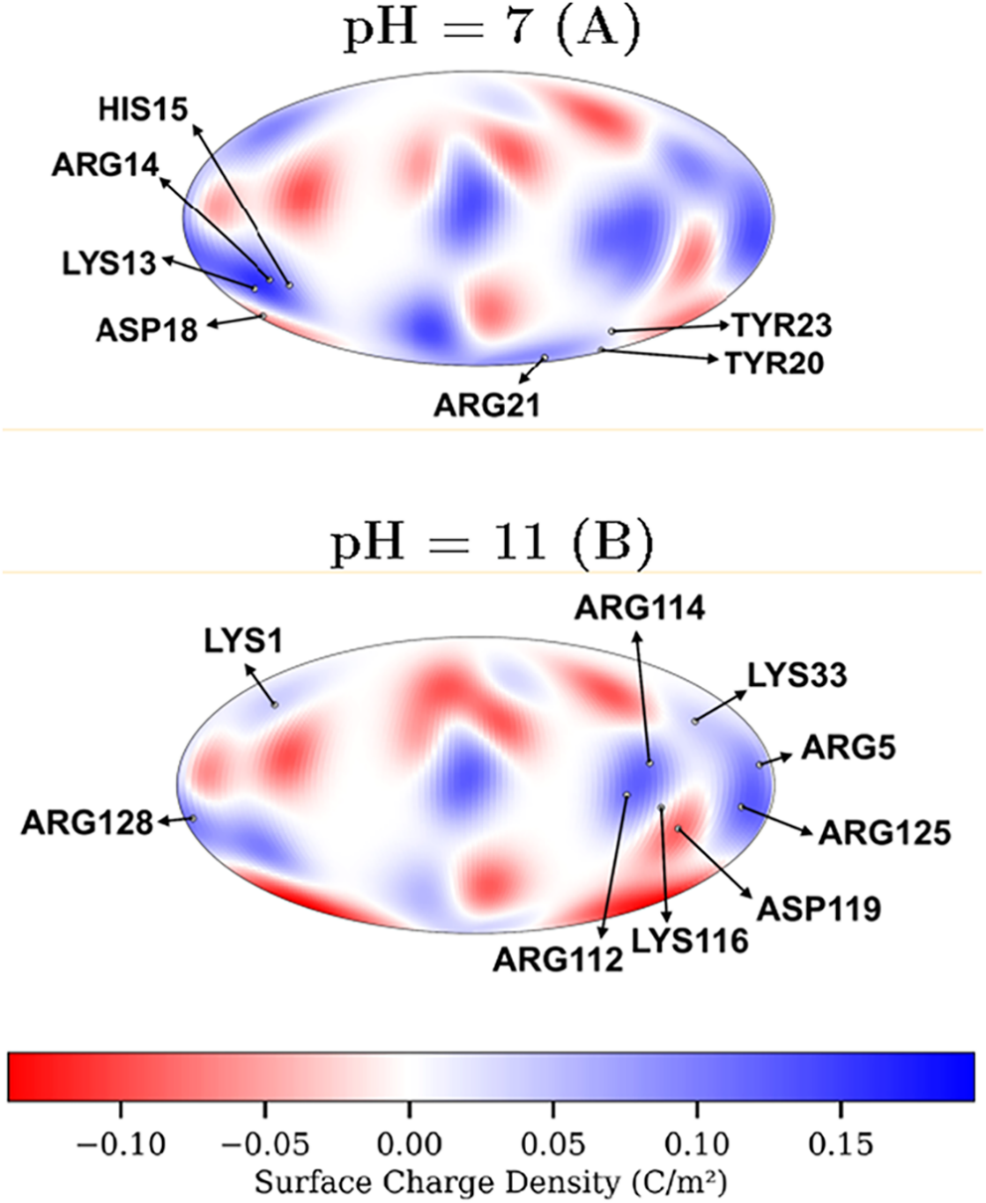
Surface charge density of HEWL in the *absence* of the CR, obtained from eq 6 and after mapping onto a plane using the Mollweide projection,^27,28,31^ for the situations of pH = 7 (A) and pH = 11 (B), as indicated. The indicated AA residues have the highest probability of being closest to the surface at each pH. The salt concentration is 0.001 M corresponding to *n*_0_ = 6.022 × 10^–7^ Å^–3^, with *N* = 30 and λ_*k*_ = 20 in eq 6.

One representative example is the positively charged patch formed by HIS15, ARG14, and LYS13, which is closer to the surface at pH = 7, see Figure 6A. The decrease in the ES attraction between this patch and the negatively charged surface—alongside the enhanced ES repulsion between the negatively charged patch formed by tyrosines and the surface—drives the orientational transition of HEWL in the absence of the CR at pH = 7.5. The AA residues TYR20 and TYR23 begin to acquire a nonzero charge already at pH ≈ 6.5, as shown in Figure 7, leading to the emergence of a large negatively charged patch at the bottom of the projection, shown in red in Figure 6B. Note that although the 2D projection displays two distinct red patches in this region, they merge into a single patch formed by the TYR20 and TYR23 residues when mapped onto a 3D spherical surface. A close spatial proximity between this large negatively charged patch and the patch containing HIS15, see Figure 6A, destabilizes the orientation in which HIS15 faces the surface. Consequently, the patch composed of ARG5, LYS33, ARG112, ARG114, and ARG125 residues, see Figure 6B, becomes preferentially oriented toward the surface because it is larger and slightly more distant from the negatively charged patch.

**Figure 7:**
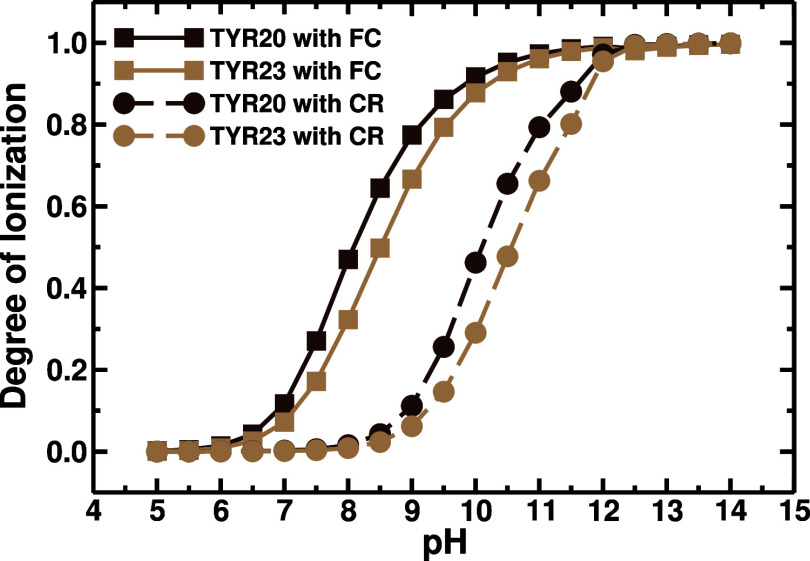
Degree of ionization of the AA residues TYR20 (dark brown) and TYR23 (light brown) as a function of solution pH. The solid (dashed) lines connecting the symbols correspond to the results obtained with the FC (CR) model. The salt concentration is 0.001 M, corresponding to *n*_0_ = 6.022 × 10^–7^ Å^–3^.

The effects of the CR are examined in Figure 8 with the help of the Mollweide projection. As the protein orientations exhibit a bimodal probability distribution, as quantified in Figure 3, the surface charge density may depend on the specific adsorption orientation when the CR mechanism is taken into account. For this reason, we separate the configurations in which either the HIS15 or the LYS33 residue is located close to the surface (at *x* < 10 Å) and calculate the surface-charge density independently for each subset of configurations. The “primary orientation” is defined as the one associated with the AA residue exhibiting a smaller average distance from the surface (see Figure 4). For instance, at pH = 7 or 8.5, the charge density projections obtained by considering only the configurations in which HIS15 residue is closer to the surface correspond to the primary orientation. As shown in Figure 8, in general, the negatively charged surface suppresses the growth of negatively charged patches on the HEWL surface, while favoring both the expansion and the enhancement of the positively charged ones, as expected from the general ES considerations.^16,17,80^

**Figure 8:**
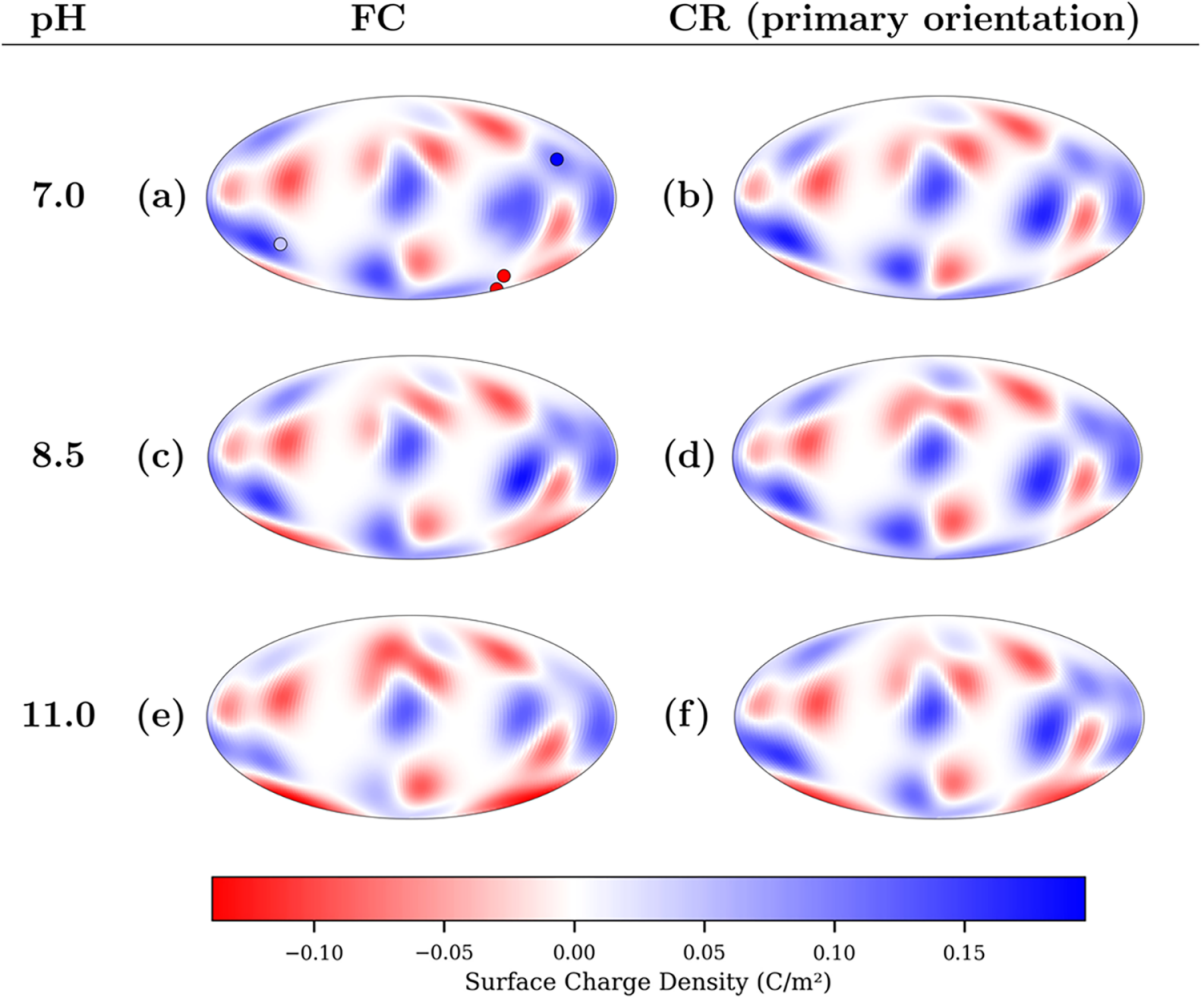
Comparison between the surface charge densities of HEWL obtained with the CR ((b), (d), and (f)) and without the CR ((a), (c), and (e)), projected onto a plane using the Mollweide projection at pH = 7 ((a) and (b)), 8.5 ((c) and (d)), and 11 ((e) and (f)), as indicated. The small circles in panel (a) show the positions of the LYS33 (blue), HIS15 (light blue), and TYR20 and TYR23 (red). Parameters are the same as in Figure 6.

At pH = 7, the surface-charge density of the adsorbed HEWL is similar for both modeling approaches (FC and CR) we utilize. At pH = 8.5, however, the presence of the negatively charged adsorbing surface triggers deprotonation of the tyrosine residues, which are responsible for the formation of the large negative patch for the FC case, see Figure 8(c). When the CR is included, see Figure 8(d), the AA residues TYR20 and TYR23 begin to deprotonate only at pH ≈ 9, as shown in Figure 7. This inhibition stabilizes the protein orientation observed at pH = 7, in contrast to the behavior obtained with the FC. Finally, at pH = 11, HEWL exhibits a pronounced decrease in the intensity of positively charged patches when the FC is assumed; see Figure 8(e). In contrast, in the presence of the CR, see Figure 8(f), the negatively charged surface partially suppresses deprotonation, so that the reduction of positively charged patches is less pronounced. Nevertheless, the deprotonation of tyrosine residues leads to the formation of a large negatively charged patch even when the CR is considered, ultimately promoting a change in the orientation of the adsorbed protein.

The magnitude of the CR response depends on the nature and the ES environment of ionizable residues. Residues with the intrinsic p*K*_a_ values close to the explored pH interval are expected to exhibit stronger CR behavior, whereas strongly acidic or basic residues mainly define the underlying ES landscape. For HEWL, the combined contribution of LYS, ASP, GLU, HIS, ARG, and of other titratable groups governs the adaptive redistribution of charge during adsorption.

Note that the results of our simulations show that the adsorption is unstable at pH = 11 for the FC case, as already shown in Figure 3. In contrast, when the CR is included, the protein remains adsorbed in a well-defined orientation. In this case, the negatively charged surface reduces the extent of deprotonation of the AA residues, yielding an average net protein positive charge of 5.4*e*_0_, as opposed to 0.5*e*_0_ in the FC case.

As the effects discussed above are of purely ES origin, it is expected that increased ES screening associated with higher salt concentrations reduces the dependence of the protein orientation on charge fluctuations. This behavior can be observed in Figures 9A and 9B, which show the population fractions of the negative and positive orientations for the salt concentrations ranging from 1 to 25 mM. The difference between the pH values at which the orientation transition occurs in the FC and in the CR models decreases at higher salt concentrations. The increase in ES screening progressively attenuates the CR effects that promote the distribution of charge patches responsible for stabilizing the orientation observed at low pH and maintained until pH ≈ 9. Therefore, the orientation transition takes place at progressively small pH values, approaching the pH at which this transition occurs with the FC.

**Figure 9:**
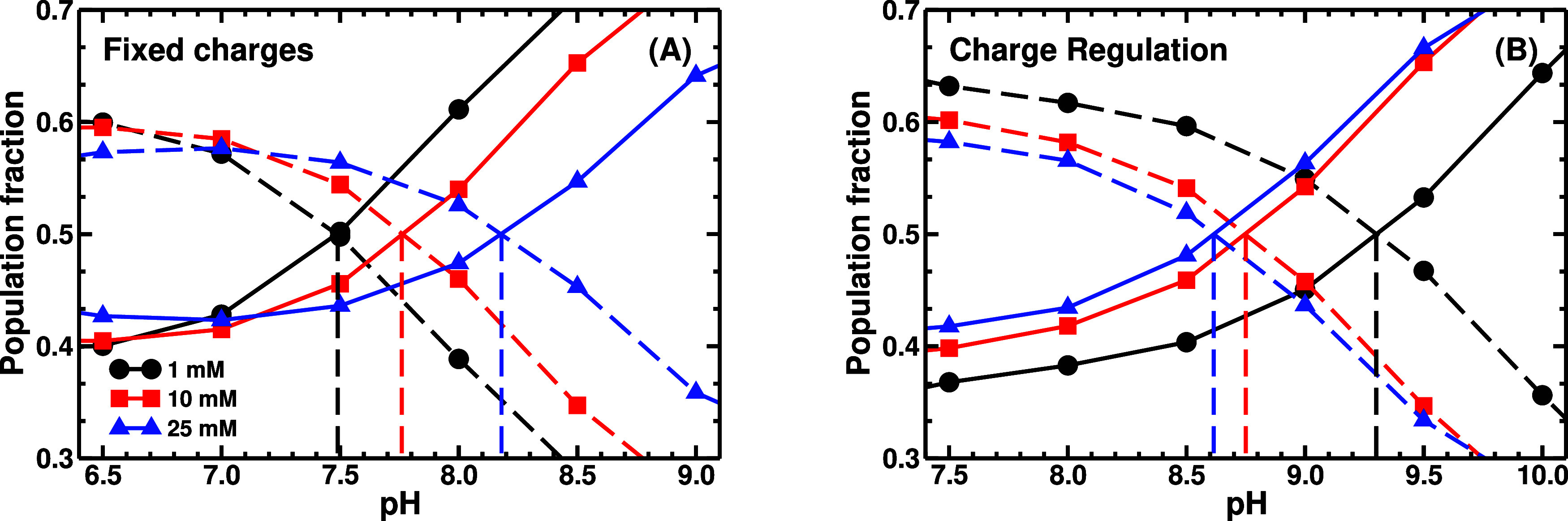
Population fractions *p_–_* (dashed lines) and *p*_+_ (solid lines) corresponding to the configurations with negative and positive angles between the short axis and the surface, respectively, as a function of pH, for three salt concentrations. Panel (A) shows the FC results, whereas panel (B) show the CR results. The transitions occur at the population fraction of 1/2, as indicated by the dotted lines in the plots. Vertical lines indicate the pH values at which the orientational inversion of the protein molecule takes place.

## 4 Conclusions

In this work, the pH-constant Monte Carlo simulations were employed to investigate the adsorption of HEWL protein as a function of the solution pH. We compared the protein orientation and its surface-charge distribution obtained in two distinct scenarios: in the first one the CR mechanism was taken into account, while in another one the charges of the AA residues were kept fixed using the FC method. Our results demonstrated that the inclusion of the CR leads to a higher degree of directionality of the HEWL adsorption onto the negatively charged surface and also induces a pH-dependent orientational transition. This transition was shown to occur at pH ∼ 7.5 in the FC-model simulations and shifts to pH ∼ 9.3 when the CR-mechanism is included in the algorithms. This difference was shown to arise from the interplay between the CR-mechanism and the formation of charged patches on the protein surface. The CR originates from the presence of a negatively charged surface, which favors the formation and stabilization of positively charged patches, while suppressing the development of the negatively charged ones. This effect enhances the protein–surface ES attraction and also stabilizes the adsorption orientation observed at lower pH values.

In comparison to our recent computer-simulation results of the properties of HEWL adsorption inside a confining pore,^25^ the novel contribution of the current investigation is the variation in the pH value at which the protein orientation changes occur when considering the CR mechanism. From a theoretical point of view, this behavior is a direct consequence of the dependence of the multipole moments of the charge density expansion on the capacitance, as discussed in ref 28 in detail.

As we consider a single protein molecule in the simulations, the present results are directly relevant to experimental conditions of *low* surface coverage. Under such conditions, the HEWL is known to adsorb with its long axis approximately parallel to the surface, corresponding to a “side-on” orientation. The arrangement is consistent with both adsorption orientations identified in our current simulations. Moreover, by considering the HEWL as a model protein, it is possible to generalize that orientational transitions may occur within the pH ranges that favor the exposure of specific protein regions containing certain binding or active sites, the sites essential for the biological function of this protein.

The results of this work can be relevant to wide practical applications, ranging from biotechnological contexts to viral evolution. Let us list here a couple of examples. First, a molecular understanding of the orientation, stability, and functionality of proteins adsorbed on charged surfaces can enhance the applications such as the functionalization of titanium implants with lysozyme—the target protein of this study—whose maximum antimicrobial efficiency would significantly reduce implantation failures.^81^

Second, in the systems involving viruses such as SARS-CoV-2, the processes of contamination with fomites, as well as viral transport and dissemination, depend on how the surface charge of solid/liquid interfaces influences the interactions with viral particles from different variants.^82–90^ In particular, based on and in parallel to the results of Pawlowski,^82,83,85^ Podgornik et al. examined^84,86,88,89^ the extent of positive-charge mutations on the spike proteins of different variants of the covid viruses, also in the presence of CR upon virus-surface interactions.^84^ These studies provided a large impulse for further scientific developments in the area, specifically in the presence of CR and in applications to virion-surface ES interactions.

Furthermore, Domingo et al. in ref 90 reported and confirmed that the spike protein on the surface of SARS-CoV-2 evolved from being highly negative in the wild-type variant to highly positive in the Omicron variant. At the same time, its charge distribution shifted from diffuse to highly localized, indicating that the intensity and the nature of the ES interactions of virions with the charged surfaces have changed throughout this surface-charge mutation-triggered evolution.^90^ An immediate benefit of such mutations would be a facilitated translocation of infectious *positively* charged virion particles though a *negatively* charged lipid membrane of a host cell upon an infection event, see refs 17,83 .

Note that the CR is intrinsically linked to the heterogeneous organization of surface charge patches for many proteins. Because the protonation changes occur locally, a charged interface may selectively stabilize or destabilize specific protonation states, thereby reinforcing or weakening localized ES domains. Such modifications alter the directional ES complementarity^62^ between the protein and the substrate and can contribute to a shift in the orientational equilibria. This physical picture is consistent with the recent molecular thermodynamic descriptions of protein adsorption under the CR conditions—including the framework proposed by Cathcart and co-workers^91^—which highlights the central role of coupled protonation equilibria in modulating the adsorption behavior over a broad range of pH values and ionic strengths.

Overall, the results presented above indicate that the synergistic interplay between the CR and charge-patch distribution is central for determining the orientation, stability, and functionality of proteins adsorbed by the ES forces on charged surfaces.

## Data Availability

The data that support the findings of this study are available from the corresponding author upon reasonable request.

## Abbreviations

HEWL: hen-egg white lysozyme
CR: charge regulation
FC: fixed charge
ES: electrostatic
AA: amino acid
